# Big data-based parathyroid hormone (PTH) values emphasize need for age correction

**DOI:** 10.1007/s40618-023-02107-2

**Published:** 2023-06-08

**Authors:** L. B. C. P. Cavalcante, C. M. Á. Brandão, M. I. Chiamolera, R. P. M. Biscolla, J. V. L. Junior, P. de Sá Tavares Russo, J. P. M. Morgado, C. M. A. de Francischi Ferrer, J. G. H. Vieira

**Affiliations:** Fleury Group, Rua Mato Grosso, 306, cj 408, Higienópolis, São Paulo, SP 01239-040 Brazil

**Keywords:** PTH value, Aging and PTH, Normal PTH, Parathyroid hormone

## Abstract

**Purpose:**

We aimed to study the relationship between aging and increased parathyroid hormone (PTH) values.

**Methods:**

We performed a retrospective cross-sectional study with data from patients who underwent outpatient PTH measurements performed by a second-generation electrochemiluminescence immunoassay. We included patients over 18 years of age with simultaneous PTH, calcium, and creatinine measurements and 25-OHD measured within 30 days. Patients with glomerular filtration rate < 60 mL/min/1.73 m^2^, altered calcemia, 25-OHD level < 20 ng/mL, PTH values > 100 pg/mL or using lithium, furosemide or antiresorptive therapy were excluded. Statistical analyses were performed using the RefineR method.

**Results:**

Our sample comprised 263,242 patients for the group with 25-OHD ≥ 20 ng/mL, that included 160,660 with 25-OHD ≥ 30 ng/mL. The difference in PTH values among age groups divided by decades was statistically significant (p < 0.0001), regardless of 25-OHD values, ≥ 20 or ≥ 30 ng/mL. In the group with 25-OHD ≥ 20 ng/mL and more than 60 years, the PTH values were 22.1–84.0 pg/mL, a different upper reference limit from the reference value recommended by the kit manufacturer.

**Conclusion:**

We observed a correlation between aging and PTH increase, when measured by a second-generation immunoassay, regardless of vitamin D levels, if greater than 20 ng/mL, in normocalcemic individuals without renal dysfunction.

**Supplementary Information:**

The online version contains supplementary material available at 10.1007/s40618-023-02107-2.

## Introduction

Parathyroid hormone (PTH) is a polypeptide produced by the parathyroid gland that plays a crucial role in the maintenance of adequate levels of calcium and phosphorus required for regular neuromuscular activity, bone mineralization, and countless other cellular and metabolic processes. The interaction between plasma levels of calcium, PTH, 1,25-dihydroxyvitamin, and phosphorus keeps calcemia within very narrow limits, even with a wide variation in food intake.

Under normal physiological conditions, PTH secretion is regulated by a complex interplay among several factors, including plasma levels and renal excretion of calcium and phosphorus, 25-hydroxyvitamin D (25-OHD) and 1–25-OHD levels, glomerular filtration rate, thyroid function, and FGF-23 (Fibroblast Growth Factor-23) [1, 2]. In clinical practice, certain medications, such as diuretics and lithium, also influence PTH levels [3, 4].

Appropriate reference values (RVs) for each specific test model of PTH directly affect the interpretation of results and, consequently, the diagnostic accuracy [5]. The diagnosis of normocalcemic hyperparathyroidism or secondary hyperparathyroidism are clinical conditions that depend on accurate PTH reference values.

Current RV levels provided by manufacturers of commercial PTH assays were obtained from assessments with a limited number of patients, primarily young and with little-known vitamin D status [6–9]. One possible reason is that recruiting and selecting volunteers in specific population segments, such as the elderly, is quite laborious. Different groups have studied PTH values in recent years. Although several studies published in the literature demonstrated a positive correlation between aging and PTH increases [6–13], most were carried out with a limited number of participants. The Fourth International Workshop on Asymptomatic Primary Hyperparathyroidism [10] highlighted that current PTH reference values might not be adequate, and assessment of larger populations with vitamin D sufficiency and age stratification was necessary.

Data mining studies, also called indirect approaches, explore large amounts of data searching for consistent patterns and have shown usefulness in special populations, such as the elderly, children, and pregnant women, and are being increasingly used [14, 15]. Therefore, we performed a real-world big data study of PTH values behavior in a Brazilian population.

We studied the values obtained for this hormone relative to age among outpatient adult individuals presenting with vitamin D sufficiency using the database of a private clinical laboratory. In addition to evaluate the correlation between PTH levels and age, we calculated the percentage of patients classified as having elevated PTH by the current reference values but who had results classified as normal using our results.

## Methods

### Subjects

We carried out a cross-sectional retrospective study and data mining with data analysis of patients who underwent parathyroid hormone (PTH) measurement in the laboratories of the Fleury Group in Brazil from 01/01/2017 to 12/31/2020. Patients between 18 and 100 years old who underwent simultaneous PTH, calcium, and creatinine measurements were included. We also included data from 25-OHD tests performed within a month of PTH collection.

All tests were performed in outpatient facilities, excluding hospital units. In the private health system in Brazil, the measurement of calcium, PTH and 25OHD is common, even in the absence of signs or symptoms of disorders of mineral metabolism. Patients with a glomerular filtration rate < 60 mL/min/1.73 m^2^ calculated using the CKD-EPI equation were excluded, and all individuals were considered nonblack for this calculation. In Grupo Fleury’s laboratories, the patient is questioned about medication use, and the information is registered in the system. Patients with a history of using lithium, furosemide, denosumab, zoledronic acid or teriparatide were excluded from the analysis. Only the first result was used if the patient had more than one PTH analysis. The cutoff values for exclusion from the study, for the dosages of 25-OHD and PTH levels, were arbitrarily selected at > 60 ng/mL and > 100 pg/mL, respectively.

Finally, all patients with 25-OHD levels ≤ 20 ng/mL, total calcium > 10.3 mg/dL were excluded.

The initial database contained 356,297 records of outpatients who underwent PTH testing between 2017 and 2020. After applying all the predetermined filters, our population sample consisted of 263,242 patients. It is interesting to point out that 5813 (1.6%) individuals of the initial database were excluded because they had total calcium above 10.3 mg/dL. All 263,242 patients had 25-OHD values ≥ 20 ng/mL (50 nmol/L) and of these, 160,660 had 25-OHD values ≥ 30 ng/mL (75 nmol/L). Then, patients were stratified by age: 18–29, 30–39, 40–49, 50–59, + 60 years (Table 1).

**Table 1:** Number of patients in each age group according to 25-OHD levels, ≥ 20 and ≥ 30 ng/mL

Age	25-OHD ≥ 20	25-OHD ≥ 30
18–29	22,169	12,448
30–39	58,846	33,460
40–49	72,783	42,357
50–59	53,239	34,457
60 +	56,205	37,938
Total	263,242	160,660

The Fleury Ethics Committee approved this study, and there was no need to use a consent form, as the survey was retrospective and only used anonymized data.

### Serum measurements

The measurement of PTH was performed by a second-generation electrochemiluminescence immunoassay (ECLIA) (Roche Elecsys, on the Cobas e602 device) on serum that uses a capture antibody directed against the C-terminus of PTH and a signal antibody directed against the N-terminus of PTH (Roche Diagnostics, Indianapolis, IN, USA). The reference values reported by the manufacturer are 15–65 pg/mL. The total coefficient of variation was 5%.

The measurement of 25-OHD was performed in serum by a competitive chemiluminescent assay (CLIA) using the DiaSorin LIAISON 25 OH Vitamin D Total assay (DiaSorin Inc., Stillwater, MN, USA) in the Liaison XL device. The total coefficient of variation was 8%.

Serum creatinine measurement was performed using a Roche CREJ2 assay (Roche Diagnostics, Indianapolis, IN, USA) on the Cobas c. The manufacturer reference values for women are from 0.50 to 0.90 mg/dL and for men are from 0.70 to 1.20 mg/dL.

Total serum calcium measurement was performed by colorimetric assay using the CA2 Calcium Gen 2 assay by Roche (Roche Diagnostics, Indianapolis, IN, USA) on the Cobas c. The manufacturer suggests as RV, for adults aged 18–60 years, 8.6–10.0 mg/dL and for adults aged 60–90 years, 8.8–10.2 mg/dL.

### Statistical analysis

Statistical analyses were performed using the R program, version 4.1.1 [16], RefineR method. Wilcoxon's multiple tests, with Benjamini–Hochberg correction, were used to investigate the differences in the mean PTH values among age groups, the relationship between PTH and serum calcium values and between PTH and creatinine values. ANOVA with Cohen's D factorial test was used to verify whether the 25-OHD values varied with age. The PTH values found in this study was calculated by bootstrapping, and a confusion matrix was used to analyze the agreement between the usual values and the values found in this study.

## Results

**Fig. 1:**
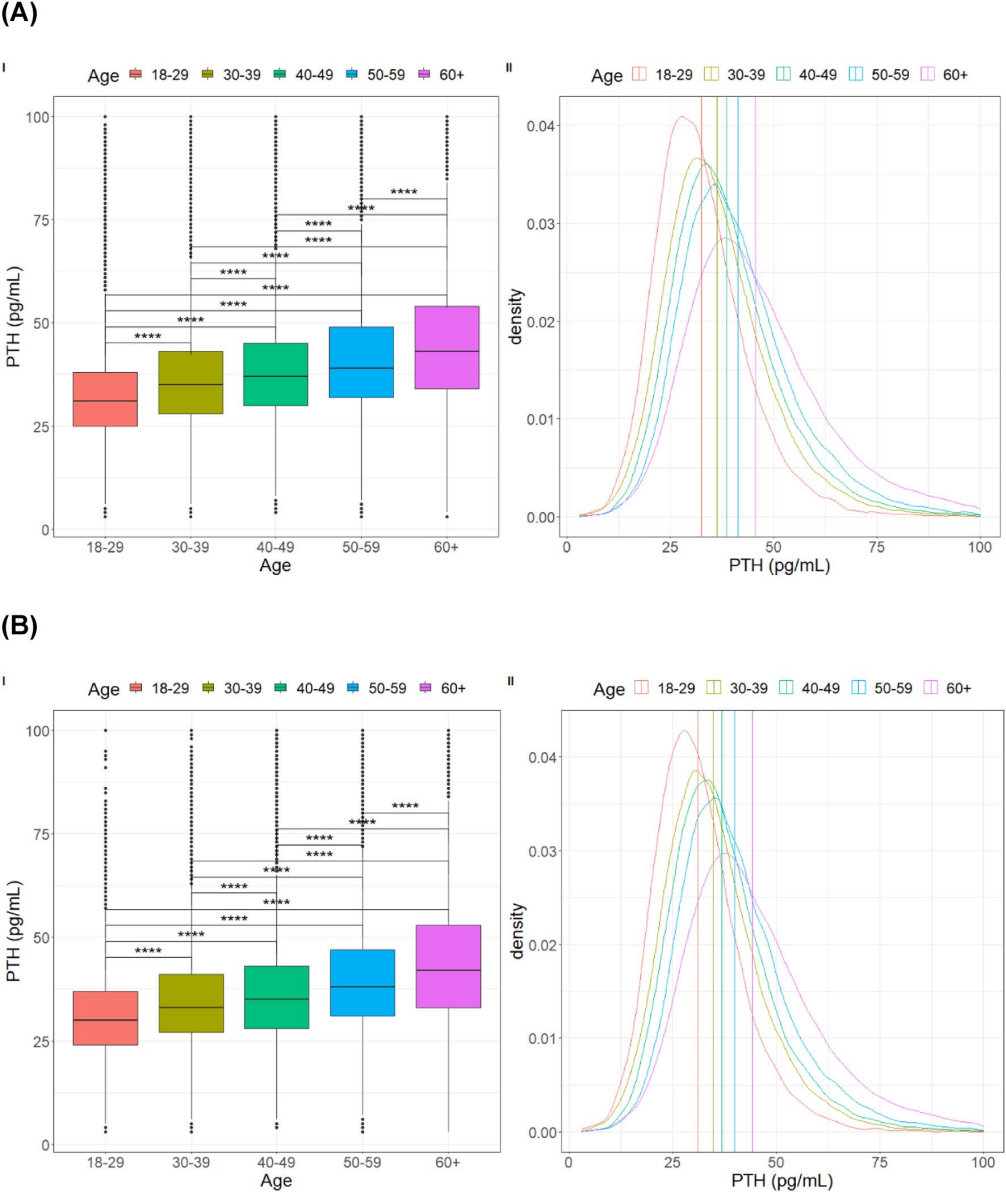
**(I)** Boxplot and **(II)** Density graph of PTH values (pg/mL) in different age groups in patients with 25-OHD above 20 ng/mL **(A)** and above 30 ng/mL **(B)**. The vertical dashed lines represent the means of PTH in the different age groups. All comparisons between groups were statistically significant (p < 0.0001)

**Fig. 2:**
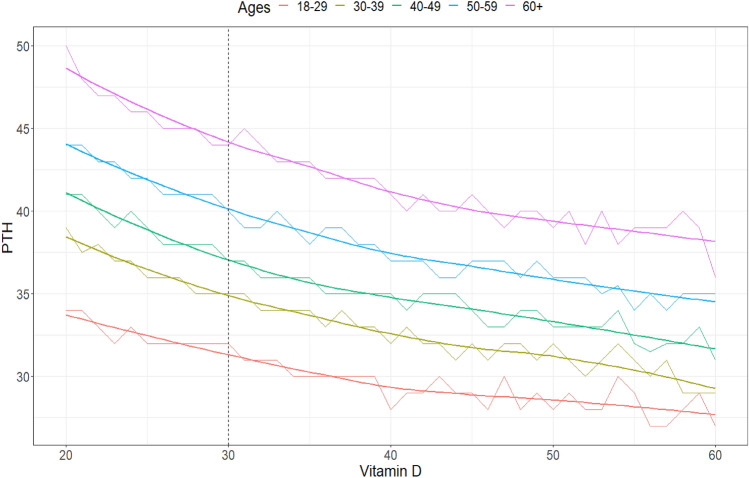
Line graph showing the relationship between vitamin D values (ng/mL) and the median PTH of individuals containing each possible analyte value for each age group. The fit line was created using the LOESS (LOcally Estimated Scatterplot Smoothing) technique. Dashed lines indicate the 25-OHD values of 20 and 30 ng/mL

PTH distributions became less dense as age increased, presenting a greater range of values. The difference in PTH values among the age groups was statistically significant (p < 0.0001), regardless of whether 25-OHD values are greater than 20 or even 30 ng/mL (Figs. 1 and 2).

In the sample studied, vitamin D levels varied slightly with age, being higher in individuals over 60 years of age, most likely due to the use of supplements with the hormone. To evaluate the importance of finding, Cohen's D test was used, and the results indicate the effects as small or insignificant.

The Mann–Whitney with Benjamini–Hochberg adjustment (alpha = 0.05) test was applied to investigate whether there was a gender difference in PTH levels. A small significant difference was observed between sexes in the younger groups, up to 39 years old, and in the group of individuals > 60 years, but Cohen’s D test pointed out that the magnitude of the effect is insignificant for all groups (Supplementary Results).

**Fig. 3:**
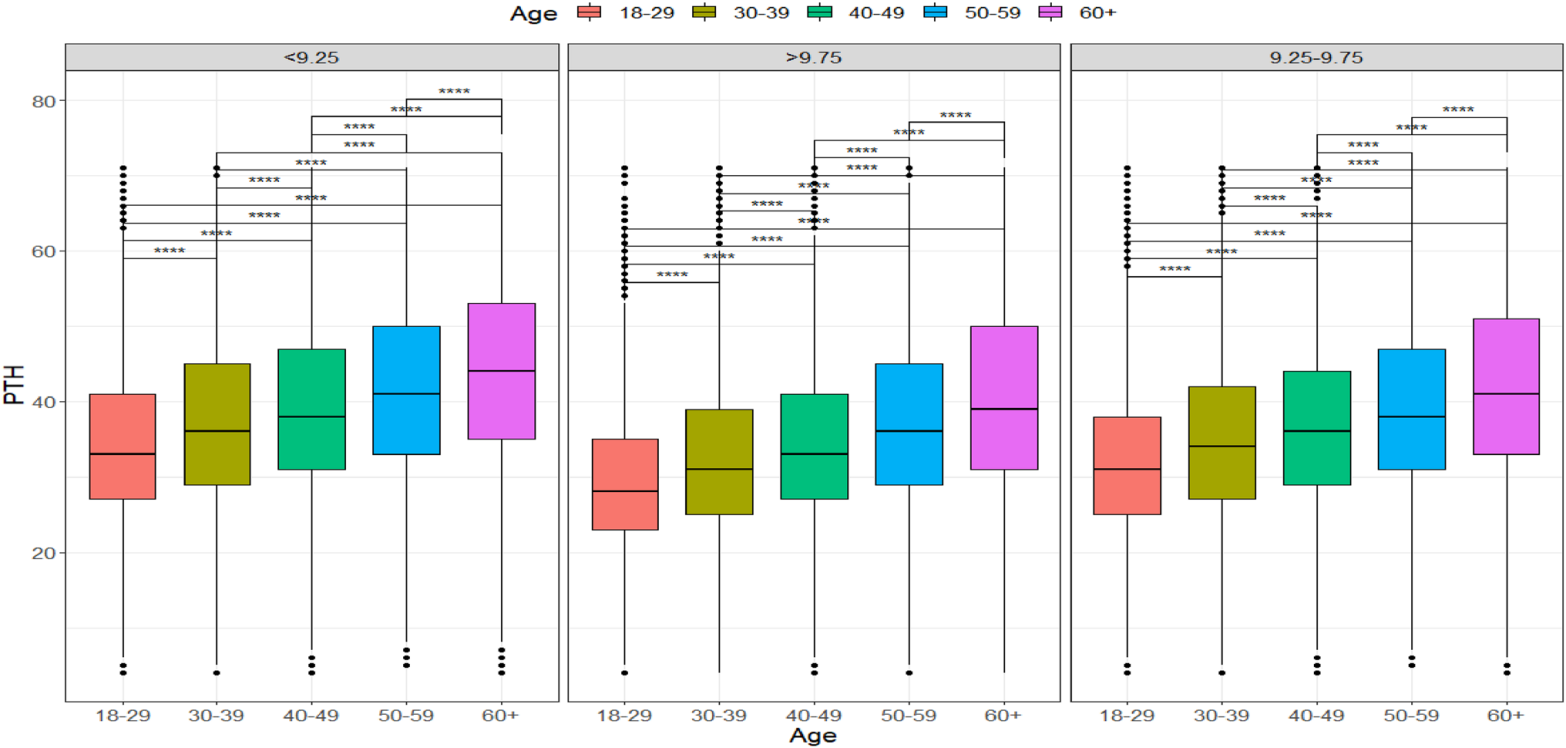
Boxplots show PTH values (pg/mL) of the total sample of individuals with 25-OHD ≥ 20 ng/mL, in different groups of calcium values (mg/dL), separated by age groups (p < 0.0001)

We also analyzed the relationship between PTH and serum calcium values using results within the laboratory's normal range of 8.6 and 10.3 mg/dL. Patients were segmented into three groups of different calcium values: less than 9.25 mg/dL, between 9.25 and 9.75 mg/dL, and above or equal to 9.76 mg/dL, and up to 10.3 mg/dL. These ranges were chosen arbitrarily to balance the number of patients in each group. We found a negative correlation (− 0.087) between calcium values and PTH. We observed that for the same range of calcium values, the PTH maintained the increase with age (Fig. 3).

We also analyzed the relationship between PTH and creatinine levels within the normal range, noting that all included patients had glomerular filtration rate > 60 mL/min/1.73 m^2^ calculated using the CKD-EPI formula. We selected patients whose creatinine values ranged from 0.7 to 1.3 mg/dL for men and from 0.6 to 1.10 mg/dL for women. Individuals were also segregated into arbitrarily chosen creatinine ranges: less than 0.75 mg/dL, between 0.75 and 0.9 mg/dL, and greater than or equal to 0.9 mg/dL. We did not observe a statistically significant difference between PTH and average creatinine values. Therefore, we concluded that PTH values are little influenced by creatinine when this parameter is within the reference range, even in elderly patients.

Then, due to the behavior of PTH according to age, we calculated reference values for PTH, obtained with the refineR algorithm, for different age groups and with specific cut-offs of vitamin D, ≥ 20 or ≥ 30 ng/mL (Table 2). In the group with 25-OHD ≥ 20 ng/mL and more than 60 years, the range was 22.1–84.0 pg/mL. We classified as false-positive results (FP) individuals with a PTH value above the upper limit of normality (ULN) by the standard RV and that were classified as normal when using the new range. A confusion matrix was performed to assess agreement between the two classifications (Table 3). When we considered patients over 60 years and with 25-OHD ≥ 20 ng/mL, the proportion of FP was 9.53%; even in individuals over 60 years and 25-OHD ≥ 30 ng/mL, we observed 7.84% of FP.

**Table 2:** Reference values for PTH (pg/mL) obtained with the refineR algorithm

	Age	C.I.	PTH
25OHD > = 20	18–29	2.5	16.4
		97.5	57.4
	30–39	2.5	18.6
		97.5	63.7
	40–49	2.5	20.1
		97.5	66.5
	50–59	2.5	21.4
		97.5	71.4
	60 +	2.5	22.1
		97.5	84.0
25OHD > = 30	18–29	2.5	15.1
		97.5	51.5
	30–39	2.5	17.0
		97.5	57.5
	40–49	2.5	18.5
		97.5	60.4
	50–59	2.5	20.9
		97.5	68.5
	60 +	2.5	21.6
		97.5	81.8

**Table 3:** Confusion matrix for patients > 60 years accounting for the agreement of the classification of patients if their PTH values are below, above, or within normal values according to the previously established ranges (10–65 pg/mL) and according to the ranges defined in Table 2

(I)	New classification			
	Under	Normal	Above	Total
Old classification				
Under	56 (0.1%)	0 (0%)	0 (0%)	56 (0.1%)
Normal	2203 (3.92%)	46,931 (83.5%)	0 (0%)	49,134 (87.42%)
Above	0 (0%)	5357 (9.53%)	1658 (2.95%)	7015 (12.48%)
Total	2259 (4.02%)	52,288 (93.03%)	1658 (2.95%)	56,205 (100%)

(II)	New classification			
	Under	Normal	Above	Total
Old classification				
Under	45 (0.12%)	0 (0%)	0 (0%)	45 (0.12%)
Normal	1317 (3.47%)	32,513 (85.7%)	(0%)	33,830 (89.17%)
Above	0 (0%)	2976 (7.84%)	1087 (2.87%)	4063 (10.71%)
Total	1362 (3.59%)	35,489 (93.54%)	1087 (2.87%)	37,938 (100%)

**(I)** Patients over 60 years and 25OHD > 20 ng/mL (**II)** Patients over 60 years and 25OHD > 30 ng/mL

## Discussion

Our study on PTH values is probably the largest in terms of the number of participants, 263,242 Brazilian adults, with an analysis controlled by serum calcium, vitamin D values and renal function.

Although RV reported by manufacturers of commercial kits for PTH assays are established based on results obtained from apparently healthy volunteers, there is no correction for other parameters that are known to interfere with the metabolism of the analyte in question, and the population sample recruited is generally younger than 60 years. Even fundamental parameters such as vitamin D status and renal function are usually absent from the analysis.

We demonstrated a correlation between aging and an increase in PTH values in a population with vitamin D sufficiency, normal serum calcium, and without renal dysfunction. Furthermore, this correlation did not differ between genders, with a slight and negligible higher value of PTH in younger women and those over 60 years of age, compared to men of the same age group. As we did not evaluate the weight of patients, perhaps this variable may influence the slight difference in PTH levels seen between men and women in certain age groups.

Previous research, using different PTH assays, observed higher upper limit values of PTH in the elderly, corroborating our findings [9, 17]. Data mining was also used by Farrel et al. [11], demonstrating that older age was associated with higher PTH concentrations among patients with optimal 25-OHD status. Age-related reference intervals showed a 63% increase in the upper and lower reference limits between the youngest (18–29 years of age) and the oldest (80 years of age or older) individuals. Delgado et al. [17] also observed that the reference intervals for PTH were age-dependent.

Touvier et al. [18] obtained PTH RV for middle-aged adults between 35 and 65 years, which were lower than those currently used. Nonetheless, we observed a gradual and significant increase in PTH values with age. Our study has some differences in terms of the evaluated subjects, PTH measurement methodology and study design. The Touvier study did not include individuals over 65 years old, while our study had a significant percentage of patients (27.16%) aged ≥ 60 years, a point that deserves to be highlighted. We did not consider the patient's weight variation or calcium intake. Another difference is methodological; we used serum instead of plasma to measure PTH, and variations in preanalytical procedures may cause variability in the results [19, 20].

Our study excluded patients with a glomerular filtration rate (GFR) < 60 mL/min/1.73 m^2^, calculated by the CDK-EPI formula. The evaluated samples come from a population in the Brazilian southeast region, highly mixed (European, black, indigenous, and Asian ancestry), with an insignificant percentage of blacks defined as children from a black father and mother. In September 2021, the National Kidney Foundation (NKF) and the American Society of Nephrology (ASN) Task Force on Reassessing the Inclusion of Race in Diagnosing Kidney Diseases released a report that outlines a new race-free approach to diagnose kidney disease. In the report, the NKF-ASN Task Force recommends the adoption of the new eGFR 2021 CKD EPI creatinine equation that estimates kidney function without a race variable [21–24].

According to our results, the RVs presented by the PTH assay manufacturer (in this case Roche) are only suitable for adults up to 49 years of age. This finding is relevant, as some patients can be classified as having elevated PTH and be misdiagnosed as normocalcemic or secondary hyperparathyroidism. In our study, in the population over 60 years of age with 25-OHD ≥ 20 mg/mL, 9.53% of patients had high PTH levels using the usual RVs but had normal PTH results using the values we found. This percentage of false positives has a great impact on the clinical evaluation of the elderly population, which can lead to misdiagnosis and even to poorly indicated surgeries for normocalcemic hyperparathyroidism.

Schini et al., in an interesting study on normocalcemic hyperparathyroidism (NPHPT), have observed greater individual variability of calcemia in patients with elevated PTH compared to control subjects. A percentage of patients with elevated PTH, according to the manufacturer’s RV, was considered normal after performing a bivariate statistical approach considering the variability of calcemia. Besides that, the authors noticed that PTH increases with age in normal control subjects, as seen in our study. This data stress the importance of discussing PTH values as a function of several variables, particularly in the diagnostic definition of NPHPT [25].

The serum PTH increase with age in both men and women has generally been attributed to an age-related decline in renal function. However, the most recent data indicate that this is not the only explanation. It has already been established that impaired intestinal calcium absorption occurs with age, partly by a decrease in the number of intestinal vitamin D receptors, leading to decreased responsiveness to 1–25-OHD [26, 27]. Additionally, PTH can be oxidized on methionine residues located at positions 8 and 18, rendering it less biologically active but still measurable by second-generation assays [28]. The presence of increased levels of reactive oxygen species (ROS) increases with age, and oxidizing PTH can also interfere with PTH receptor signaling and trafficking [29]. These two effects can contribute to the finding of higher PTH levels with increasing age.

It is not yet clear whether the increase in PTH with age is just an adaptative phenomenon with no harmful consequences or is a detrimental event. Several studies have suggested a correlation between PTH levels and mortality rates in the elderly. Domiciano et al. [30] observed that PTH levels and low bone mineral density, are independent risk factors for mortality in older adults. This may result from the noncalcemic effects of PTH, such as atherogenesis by vascular calcification, left ventricular hypertrophy, and direct effects on vascular smooth muscle cells. Suriyaarachchia et al. [31] demonstrated that older persons with high levels of serum PTH have a higher prevalence of falls and fractures, in addition to a strong association with osteosarcopenia, even in the absence of vitamin D deficiency or impaired renal function.

The large population sample, 263,242 Brazilian adults, and the adjustment for related parameters are the greatest strengths of our study, but there are also some limitations. First, the lack of medical history of the participants was minimized by the exclusion of patients who reported using medication known to influence PTH values, such as loop diuretics, lithium, denosumab, zoledronic acid and teriparatide, and the use of data from outpatient collections only. In our country, in medical private practice, the criteria for requesting exams are flexible and patients with no history of metabolic bone diseases routinely perform PTH measurement.

Another limitation could be the measurement of 25-OHD levels within a month of PTH collection. However, we believe that there is little impact because of the total of 263,242 individuals, just 20,112 reported using cholecalciferol continuously, which represents only 7.64% of the sample.

We also do not have information on patients’ weight, body mass index, dietary calcium intake or urinary calcium excretion, which are which are variables affecting hormonal outcomes [18]. As hypercalciuria and intestinal calcium malabsorption were not evaluated in our study, and we included patients with PTH values up to 100 pg/mL, a certain number of cases of Secondary Hyperparathyroidism, due to these causes, were probably included in our sample.

Finally, as our population consisted of approximately 263,000 people with outpatient follow-up and in a private laboratory, some patients with disorders of mineral metabolism would have little impact on the overall analysis.

Another issue to be addressed when discussing PTH value is the variability between different assays. Souberbille et al. [20] compared the PTH results obtained through 15 different assays from the same serum sample and showed discrepant values. Furthermore, other studies have shown variation in PTH results measured by the same assay when serum and plasma samples are used [32]. Additionally, significant differences exist in values obtained with assays provided by different manufacturers [33]. This aspect can be even more important when 3rd generation assays are used, since they report lower values due to their specificity, limited to the bioactive PTH form [34]. Thus, we must emphasize that we cannot generalize our data to all assays, and it only applies to the 2nd Generation Roche assay, measured on a Roche Cobas e602 platform, using serum samples.

In conclusion, we showed that age influences PTH values and that clinical laboratories should establish specific RVs for elderly individuals according to the PTH assays used. This effort will help in understanding the role of PTH in aging and in obtaining an accurate diagnosis of metabolic bone diseases.

### Supplementary Information

Below is the link to the electronic supplementary material.

Supplementary file1 (DOCX 119 KB)

## Data Availability

Data are available in: https://grupofleury.sharepoint.com/:f:/s/ProjetosdeDados/EoU888nXfUpFnFvRd6rWbFwBadsaWnfMk1rKJXtV1F26fg. This link will be available for a limited period of time, only for peer review and referee analysis. This dataset will not be used in any other paper or study.
