# Supplementary material for: Big data-based parathyroid hormone (PTH) values emphasize need for age correction
Source: J Endocrinol Invest. 2023 Jun 8;46(12):2525–33. doi: 10.1007/s40618-023-02107-2 (PMC10632255; doi:10.1007/s40618-023-02107-2)

**Suplementary Material**

**Figure A**. **I)** Significance of the Mann-Whitney test to evaluate the difference between PTH values and age groups in each sex. **II)** Cohen's D test to verify the magnitude of the effect of the difference between genders by age groups. **III)** Density of PTH values by ages

**I)**


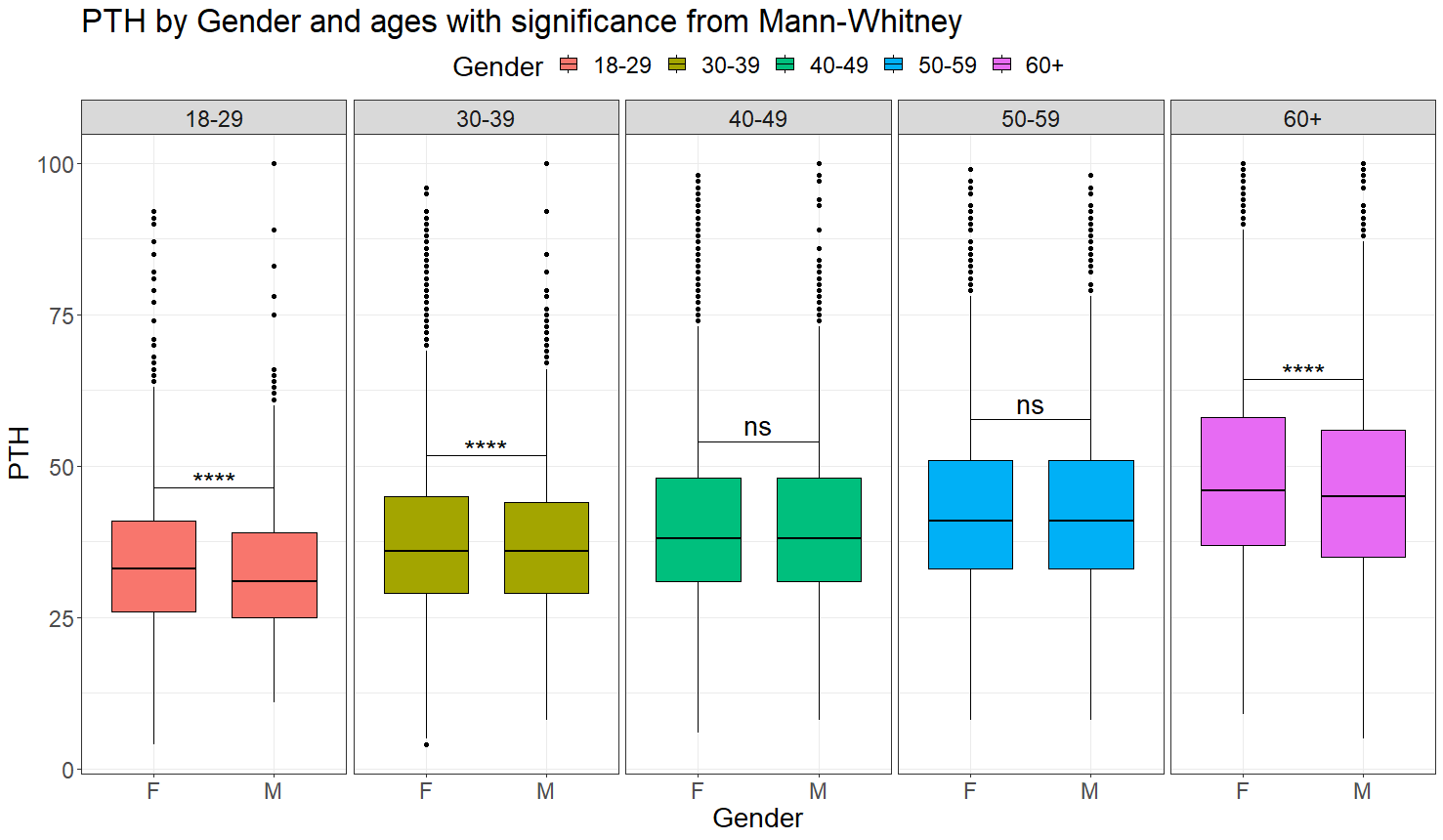


**II)**

| **Age** | **Group 1** | **Group 2** | **Effect** | **#Pacients 1** | **#Pacients 2** | **Magnitude** |
| --- | --- | --- | --- | --- | --- | --- |
| 18-29 | F | M | 0.129 | 15479 | 6690 | negligible |
| 30-39 | F | M | 0.109 | 41896 | 16950 | negligible |
| 40-49 | F | M | 0.0148 | 51377 | 21406 | negligible |
| 50-59 | F | M | -0.0108 | 37049 | 16190 | negligible |
| 60+ | F | M | 0.0694 | 37112 | 19093 | negligible |
| **III)** |  |  |  |  |  |  |


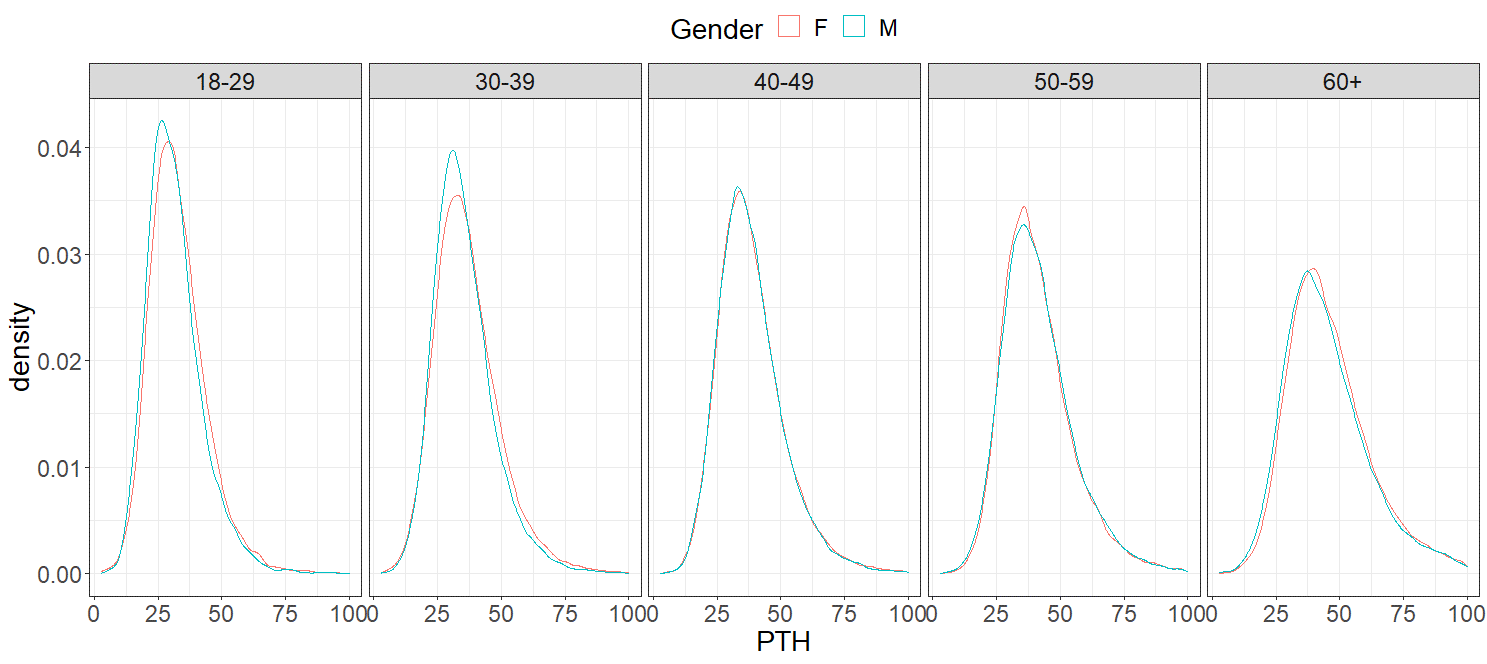

Supplement: Supplementary file 1 — Supplementary file1 (DOCX 119 KB) [file 40618_2023_2107_MOESM1_ESM.docx]
